# Postoperative Pain Control by Local Infiltration Analgesia and Peripheral Nerve Block in Primary Prosthetic Total Knee Arthroplasty

**DOI:** 10.5041/RMMJ.10476

**Published:** 2022-07-31

**Authors:** Alexey Vladimirovich Lychagin, Andrey Anatolyevich Gritsyuk, Nahum Rosenberg

**Affiliations:** 1 Department of Traumatology, Orthopedics and Disaster Surgery, I.M. Sechenov First Moscow State Medical University, Moscow, Russian Federation; 2 CEO, Sheltagen Medical Ltd, Atlit, Israel; 3 Medical Director, Specialist Center, National Insurance Institute, North Branch, Haifa, Israel

**Keywords:** Knee arthroplasty, local infiltration analgesia, nerve block, pain control

## Abstract

**Background and Objective:**

Postoperative (post-op) pain control has an important impact on post-op rehabilitation. The logistics of its maintenance challenge the effect of peripheral nerve block on post-op pain control, with the risk for post-op complications. We hypothesized that perioperative use of local infiltration analgesia (LIA) is comparable to post-op pain control by peripheral nerve block.

**Materials and Methods:**

We evaluated three groups of patients treated with primary total knee arthroplasty (TKA) due to symptomatic end-stage osteoarthritis with post-op pain control by LIA (LIA group, *n*=52), femoral plus sciatic nerve block (FSNB) (FSNB group, *n*=54), and without local or regional analgesia as controls (Control group, *n=*53). The primary outcome variable was the post-op pain level intensity as measured by the visual analog scale (VAS). Secondary outcome variables were knee function measured by the Knee Society Score (KSS) and the quadriceps muscle strength recovery profile.

**Results:**

Up to 4 hours post-op, pain intensity was significantly lower in FSNB patients (*P*<0.05). This effect of the peripheral nerve block on the pain level disappeared 6 hours post-op. The LIA and FSNB patients showed a significant decrease in pain intensity on days 2 and 3 post-op (*P*<0.05) with no mutual differences (*P*>0.05). This effect disappeared on day 4 post-op (*P*>0.05). The KSS score showed similar significant improvement of functional abilities (*P*<0.001) in all three groups. There was no difference in KSS scores among the groups 6 months after surgery (*P*>0.05). Quadriceps muscle recovery profile was similar in the LIA and Control groups, but significantly poorer in the FSNB group (*P*<0.001).

**Conclusion:**

The value of very short-term and improved pain relief of post-op FSNB over LIA of the surgical wound should be carefully weighed against its cost, logistics, and potential complication threat.

## INTRODUCTION

Total knee arthroplasty (TKA) is a standard and usually highly effective surgical procedure for treating advanced symptomatic knee joint degeneration.^1^,^2^ However, the clinical outcome is suboptimal in about 20% of treated patients,^3^,^4^ as repeatedly reflected in different reports over the past two decades.^5^,^6^ One of the common undesired sequelae of TKA is chronic pain.^7^

The preferred surgical anesthesia in TKA depends on the anesthetist’s skill and experience and is mainly based on general, regional, or combined methods,^8^,^9^ with advantages and disadvantages of each.^10^,^11^

Surgical anesthesia has an essential impact on postoperative (post-op) rehabilitation,^12^ partially affecting post-op pain control. Postoperative pain control is based on oral and parenteral pharmacological means. Peripheral nerve block is an additional widely used method for post-op pain control.^13^ However, its effect on post-op pain control is challenged by maintenance logistics and a subsequent risk for post-op complications due to mishandling of inserted catheterization devices.^14^–^16^ Therefore, prolonged catheterization techniques for intra-articular and peripheral nerve blocks have been developed to reduce the risk of wound infection. However, their significant advantage is debatable.^17^–^19^

Accordingly, local infiltration analgesia (LIA) was suggested as a relatively simple method for post-op pain relief.^20^–^25^ Potentially, this method should reduce the rate of hemodynamic or neurological side effects and is technically and logistically less complicated and, therefore, more cost-effective.^26^,^27^ The effectiveness of peripheral nerve block compared to other modalities of post-op pain control is the subject of ongoing debate^28^; however, such information is of high importance for improving post-op pain control and its relation to the functional rehabilitation of patients.^29^–^31^

There is some evidence indicating that LIA is superior to femoral plus sciatic nerve block (FSNB) in preserving the functional activity of the quadriceps muscle.^32^ Thus, we hypothesize that perioperative LIA for pain control would be comparable to post-op peripheral nerve block and, therefore, might be advantageous due to its relatively easy and safe mode of implementation.

Accordingly, this study aimed to compare patients’ post-op functional ability after TKA with perioperative pain control by LIA of the surgical wound versus post-op peripheral FSNB.

The primary outcome variables were post-op pain level intensity measured by the visual analog scale (VAS). The secondary outcome variables were knee function measured by the Knee Society Score (KSS) and the quadriceps muscle strength recovery profile.

## MATERIALS AND METHODS

The study was conducted according to the guidelines of the Declaration of Helsinki and approved by the Institutional Review Board (Ethics Committee) (#129, 2015). All participating patients signed an informed consent.

### Study Group

Patients treated with primary TKA due to symptomatic end-stage osteoarthritis of the knee between January 2016 and November 2017 were recruited for this study. All patients were treated in the same tertiary referral academic medical center (Department of Traumatology, Orthopedics and Disaster Surgery, University Hospital No. 1, I.M. Sechenov First Moscow State Medical University, Moscow, Russian Federation). The inclusion criteria were: age 40 to 85 years old, and radiologically confirmed diagnosis of knee osteoarthritis (stage 3–4 on the Kellgren–Lawrence scale^33^).

The exclusion criteria were: body mass index (BMI) <20 or >35 kg/m^2^, high anesthetic risk (above 3 points according to the ASA Physical Status Classification System assessment for pre-anesthesia medical comorbidities^34^), history of thromboembolic and infectious complications, uncontrolled diabetes mellitus, chronic treatment by steroids, anemia, thrombophilia, allergies to local anesthetics, and ligament insufficiency in the knee joint.

Patients were randomized into three groups using dedicated software: a perioperative LIA group, post-op FSNB group, and Control group. The randomization results were reported to the operating surgeon and the anesthetist immediately before the operation. Health care personnel recording patient data following surgery were blinded to the type of pain medication used (LIA, FSNB, or Control).

The surgeries were performed by two experienced surgeons (authors A.V.L. and A.A.G.). All patients were anesthetized using subarachnoid anesthesia with intravenous sedation.

The LIA group received local infiltration analgesia, administered into the projection of a skin incision. After implantation of the endoprosthesis, a periarticular infiltration of the same local anesthetic was implemented.

The FSNB group received ultrasound-guided nerve block (FSNB) of the treated limb following surgical wound closure.

The Control group was treated for post-op pain control according to standard protocol with pharmacological, parenteral, and oral agents.

### Anesthetic Protocol

The preoperative premedication was identical for all study groups: 12 hours preoperatively, the patients received oral benzodiazepine (1 mg, phenazepam, Valenta Pharm A.O., Moscow, Russia). One hour before surgery, 1 mL of phenazepam solution was administered intramuscularly. Thirty minutes before skin incision, 1 g of cephalosporin antibiotic (ceftriaxone, Virend International LLC, Moscow, Russia) was administered intravenously and repeated twice, 12 and 24 hours after surgery for perioperative infection control. Ten minutes before skin incision, 10 mg/kg intravenous tranexamic acid (Tranexam, FSUE Moscow Endocrine Plant, Moscow, Russia) was given for intraoperative bleeding control.

All patients in the study received subarachnoid anesthesia and intravenous sedation. Before the subarachnoid puncture, the patient was injected intravenously with 2.5–5 mg diazepam (WPG Pharma GmbH, Heidelberg, Germany) and a single dose of 8 mg dexamethasone (KRKA d.d., Novo Mesto, Slovenia). The subarachnoid puncture was administered at the L3–L4 level with a 25–27 G needle, with the patient in the sitting position. After obtaining cerebrospinal fluid flow, an isobaric 0.5% solution of bupivacaine (Bupivacaine-binergia, Binergia Zao, Balashikha, Russia) was administered intrathecally (2.5–3 mL). Intraoperative sedation was provided by intravenous infusion of propofol (1 g/50 mL), with 40 mg induction anesthesia every 10 seconds until clinical signs of anesthesia were evident (total dose, 2–2.5 mg/kg); anesthesia was maintained by administering 4–12 mg/kg/h.

### Surgical Technique

The surgical approach was performed along the anterior knee midline, dissecting the capsule and the medial patella retainer with its lateral dislocation. No tourniquet was used. Hemostasis was maintained using electrocoagulation. The standard arthroplasty technique was used: NexGen^®^ Total Knee System Cemented Zimmer^®^ (Zimmer Biomet, Warsaw, IN, USA) or DePuy^®^ P.F.C.^®^ SIGMA Total Knee System (DePuy Synthes, Warsaw, IN, USA) with cruciate-retaining implants with Prolong Highly Crosslinked Polyethylene fixed bearing. Before cementing, a single bolus dose of 8 mg dexamethasone was administered intravenously. The patella was not resurfaced, aside from osteophyte removal and circular denervation. The wound was closed in layers using non-absorbable sutures (Ethibond green M5 4×75 cm, taperkat needle) over the joint capsule without leaving drainage. Subcutaneous tissue was closed with absorbable sutures, and skin was closed with metal braces (Covidien Appose LRC 35W Single Use Skin Stapler, Covidien, Minneapolis, MN, USA). The wound was covered with a post-op sterile self-adhesive dressing (Cosmopor E, 20×10 cm, Heywood, UK).

All patients received an intraoperative infusion of 1500–2000 mL of 3:1 crystalloid and colloidal solution.

Patients in the LIA group were treated by a 1% lidocaine hydrochloride 20–40 mL (200–400 mg, Armavirskaya B.F., Moscow, Russia) infiltration into subcutaneous tissue at the skin incision of the surgical site. Following cementing of the prosthetic device, all the adjacent periprosthetic soft tissues were infiltrated circularly with a solution containing 100 mL of 0.2% ropivacaine (Fresenius Kabi Deutschland GmbH, Hamburg, Germany) and 0.5 mL adrenaline (1:10000) diluted with isotonic saline, for a total volume of 150 mL.

Patients in the FSNB group were treated as follows: an anesthetist administered a peripheral nerve block immediately following surgical wound closure and dressings to the femoral and sciatic nerves under ultrasound guidance. For this purpose, we used a single dose of 100 mg of bupivacaine (40 mL of a 0.25% solution) with 0.5 mL of adrenaline (0.1 mg/mL).

### Postoperative Protocol

During the first 24 hours post-op, all patients received a standard systemic multimodal analgesia, which included a combination of NSAIDs: ketoprofen (CJSC Organika, Novokuznetsk, Russia) 100 mg three times a day, and paracetamol (FarmaSino Pharmaceuticals Ltd, Nanjing Jiangsu, China) 1 g three times a day; and an intramuscular opioid analgesic, tramadol (KRKA d.d., Novo Mesto, Slovenia) 100 mg once a day. The dosage and frequency of these medications were adjusted according to the patient’s post-op pain intensity.

All patients received subcutaneous injections of low-molecular-weight heparin (LMWH, 0.2 mL, 2000 IU; Clexane, Sanofi-Aventis, Paris, France) 6 hours after surgery, which was repeated at 24-hour intervals for up to 3 days after surgery. Thereafter, and if there was no post-op bleeding, 10 mg of oral rivaroxaban (Xarelto, Bayer, Mississauga, Ontario, Canada) was administered for 30 days to prevent thrombosis.

The rehabilitation protocol included initiating passive knee movement 2 hours after surgery. Active exercise and gradual ambulation were encouraged 24 hours after surgery. Walking with limited weight-bearing (using crutches) was encouraged up to 3 weeks after surgery, after which full weight-bearing when walking was allowed.

Before surgery and during the first 5 days post-op, knee pain intensity was recorded using an 11-point Visual Analog Scale (VAS), with 0 indicating no pain and 1–10 points indicating the severity of pain, with 10 being the most severe. The VAS values were monitored every 2 hours during the first 6 hours post-op and at 12 and 24 hours post-op, then reevaluated every 24 hours for up to 5 days post-op.

Passive range of motion (ROM) in the operated knee joint was recorded before surgery and 4 days, 2 weeks, and 6 months after surgery. Measurements were taken using a manual goniometer with patients in a supine position.

The clinical and functional status of patients was assessed using the Knee Society Score (KSS) before surgery and 6 months after surgery. The KSS is a 100-point scale based on estimating pain, alignment, ROM, and stability of the knee joint. Scores of 80 and above, 70–79, 60–69, and below 60 points are defined as excellent, good, fair, and poor function, respectively.^35^

The progress of quadriceps muscle strength recovery was determined according to the recommendations of the Medical Research Council (United Kingdom). In particular, the Straight Leg Raise (SLR) test was used; scores ranged from 0 to 5, where 0/5 indicated no muscle contraction and 5/5 indicated maximal muscle contraction. This test was recorded daily for the first 5 days post-op.^36^

Patient records were examined for possible complications associated with analgesics or surgical interventions during the first 7 post-op days, e.g. the incidence of neurological complications, cardiovascular complications, falls, knee infections, and prosthesis malalignment that required revision surgery.

### Statistical Analysis

The VAS was the primary outcome assessed in all three study groups.

Normally distributed continuous variables were compared using a one-way ANOVA and post hoc test for more than two variables. Proportions were analyzed by the Pearson chi-square or the Fisher exact test. Statistical significance was set at *P*<0.05 for all statistical tests.

Statistical analysis was performed using the SPSS Statistics 22.0 statistical software (SPSS Inc., Chicago, IL, USA).

## RESULTS

### The Final Study Group

A total of 180 patients met the inclusion criteria and agreed to participate in the study. After randomization, 60 patients were initially assigned to the three groups: perioperative LIA group, post-op FSNB group, and Control group.

After the surgery, 21 patients were removed from the study: 5 patients due to wound infection, 1 patient due to periprosthetic fracture, 3 patients due to systemic complications, and 12 patients were lost to follow-up due to failure to attend the follow-up clinic (5, 3, and 4 patients, from the LIA, FSNB, and Control groups, respectively). These last-mentioned 12 patients had no early post-op complications during the hospitalization period. Therefore, a total of 159 patients were analyzed (88% from the initial study group), 23 men (14.5%) and 136 women (85.5%). The average age was 68 years (range 40–85 years). Analysis of the demographic characteristics of each group revealed no statistically significant differences regarding BMI and the American Society of Anesthesiologists (ASA) Physical Status Classification System, which grades for indications of pre-anesthesia medical comorbidities (Table 1).

**Table 1:** Preoperative Demographics.

Demographics	LIA Group (*n*=52)	FSNB Group (*n*=54)	Control Group (*n*=53)	*P* Value
Age (y)*	67.1±9.3	68.8±6.9	67.8±8.5	0.548

Gender: Female/Male†	44/8	47/7	45/8	0.927

Mean BMI±SEM (kg/m^2^)	32.5±2.5	32.1±2.1	32.2±2.1	0.568

ASA†				
I (*n*/%)	4/7.7	5/9.3	6/11.3	0.954
II (*n*/%)	23/44.2	25/46.3	25/47.2	
III (*n*/%)	25/48.1	24/44.4	22/41.5	

* Analyzed by one-way ANOVA.

† Analyzed by Pearson chi-square or Fisher exact test.

ASA, American Society of Anesthesiologists; BMI, body mass index; *n*, number of patients; y, years.

### Pain Intensity and Range of Motion

**Figure 1:**
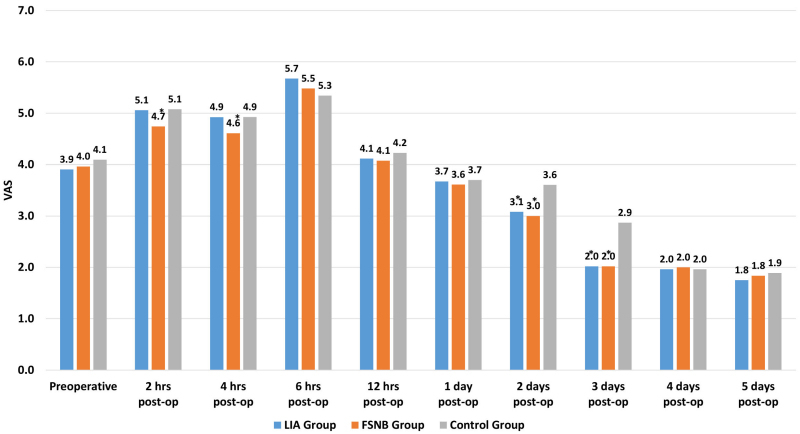
Study Group Profiles of Pain Intensity Based on the Visual Analog Scale (VAS) after Total Knee Arthroplasty. The FSNB patients experienced significantly improved short-term pain relief for 4 hours postoperatively. Both FSNB and LIA patients experienced significantly reduced pain intensity on days 2 and 3 postoperatively. * *P*<0.05.

The preoperative pain intensity was similar in all study groups (Figure 1). Up to 4 hours post-op, pain intensity was significantly lower in the FSNB group (*P*<0.05, ANOVA; Figure 1). This advantageous FSNB effect on the pain level disappeared 6 hours post-op. Compared to the Control group, both the LIA and FSNB-treated patients experienced a significant decrease in pain intensity on the second and third post-op days (*P*<0.05, ANOVA; Figure 1) with no mutual differences (*P*>0.05, ANOVA, Figure 1). This effect disappeared on the fourth post-op day (*P*>0.05, ANOVA, Figure 1).

There was no significant difference in the ROM of the operated knees before surgery (mean 107°±1° SEM, all, for the LIA, FSNB, and Control groups, respectively; *P*>0.05, ANOVA) and at 6 months follow-up (mean 110°±1° SEM, all, for the LIA, FSNB, and Control groups, respectively; *P*>0.05, ANOVA); thus, there was no difference in ROM among the groups and no change when compared before and 6 months after surgery.

The KSS score for analyzing functional ability showed similar significant functional improvement (*P*<0.001) in all study groups, i.e. poor function preoperatively and excellent function 6 months post-op. Notably, no difference in KSS scores among the groups before and 6 months post-op were noted (preoperative mean 51±1 SEM, 51±1 SEM, and 55±1 SEM for the LIA, FSNB, and Control groups, respectively [*P*>0.05, ANOVA]; and post-op mean 84±1 SEM, all, for the LIA, FSNB, and Control groups, respectively [*P*>0.05, ANOVA]).

**Figure 2:**
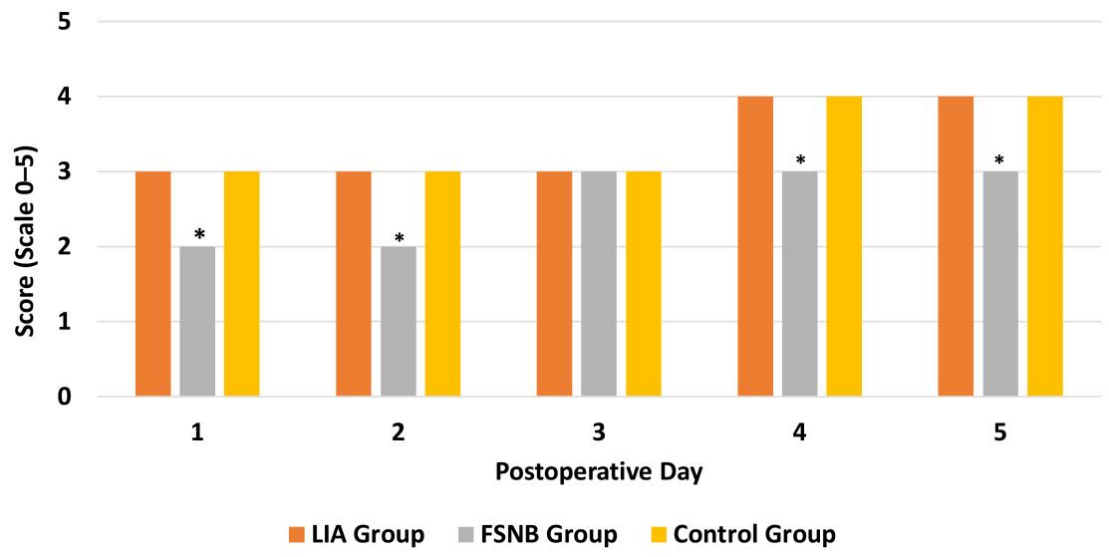
Study Group Profiles of Postoperative Muscle Recovery after Total Knee Arthroplasty. Pain was measured on a scale of 0–5. * Significantly poorer muscle recovery profile (*P*<0.001).

Quadriceps muscle recovery profile was similar in the LIA group and Control group, improving from a mean 3/5 score on the first 3 post-op days to 4/5 scores on days 4 and 5 after surgery. This profile was significantly poorer in the FSNB group (*P*<0.001, ANOVA), with a mean 2/5 score the first 2 days post-op, which increased to a mean 3/5 score from the third to fifth post-op days (Figure 2).

## DISCUSSION

The post-op pain intensity was more effectively reduced in the first 4 hours after surgery in the FSNB group than in the LIA and Control groups. Both FSNB and LIA had no significant effect on pain when it peaked 6 hours post-op and at 12 hours and 1 day. Only at post-op days 2 and 3 did both FSNB and LIA provide significantly reduced pain relief compared to controls. From post-op day 4, the pain levels in all study groups became low and bearable (VAS values of ≤2). Therefore, LIA and FSNB provided earlier opportunities for effective rehabilitation commencement.

This observation supports the hypothesis that local post-op analgesia enhances the recovery pace of the quadriceps femoris muscle function, which appears to be significantly faster in LIA-treated patients than in FSNB-treated patients, and with no significant difference compared to controls. This phenomenon can be explained by the lack of neurotrophic effect on the muscle due to several hours of FSNB.

Interestingly the described attempts of post-op pain control modulation by LIA and FSNB were not reflected in the final functional outcome, when the expected rehabilitation should be finalized, i.e. 6 months post-op, as reflected by similar KSS scores and knee ROM in all three study groups.

These results show that peripheral nerve block provides better but shorter-term pain relief in the first 4 hours post-op, with an undesired delay in muscle recovery. Although both FSNB and LIA cause a more significantly delayed analgesic response in the initial 5 days post-op, they do not alter the final functional outcome after TKA surgery.

The results of this study are substantiated by similar recently published results on effective post-op pain control and muscle recovery following perioperative LIA in primary TKA.^37^ Also in line with previous studies, we showed a mutually indistinguishable effect of LIA and FSNB 24 hours after surgery,^38^ which was superior to the effects of post-op epidural opioids and intrathecal analgesics.^39^,^40^ Since the systemic toxicity of LIA can affect up to 11% of patients, the post-op monitoring of LIA patients cannot be ignored.^41^ A similar observation was made by Tian et al.; their retrospective TKA study showed that the short-term analgesic effect of LIA was similar to that of nerve block and reduced the need for opiate supplementation, and that LIA and nerve block had similar complication rates.^42^ There is also evidence of marginal early post-op pain management improvement in patients treated by LIA as compared to peripheral nerve block, when evaluated 24 hours post-op.^43^ However, none of these reports provides combined pain intensity data with hourly post-op measurements together with long-term functional follow-up.

Regarding post-op muscle strength, our results were similar to those of Berninger et al. They showed better restoration of muscle strength in the first 5 days post-op in LIA-treated patients compared to those receiving post-op peripheral block.^44^ This may indicate that perioperative pain control by LIA has a more effective rehabilitation potential in patients than post-op FSNB.^44^ However, the significant advantage of FSNB for better pain reduction in the initial post-op hours should not be overlooked, especially in patients with lower pain tolerance. A prospective study by Tanikawa et al. showed less pain experienced by patients treated with the sciatic block compared to LIA. However, this advantageous effect disappeared after the first 3 hours post-op, similar to our observations of the LIA versus FSNB groups.^45^

The findings of all the above-mentioned studies were quite similar to our own findings regarding the effectiveness of pain control of LIA versus FSNB.^17^ Femoral plus sciatic nerve block has a marginal advantage in reducing pain during the initial 4 hours post-op, but muscle strength improvement is better during the first 5 days post-op in LIA patients (Table 2).

**Table 2:** Selected Reports on Pain Control Efficiency after Knee Prosthetic Replacement by Local Infiltration Analgesia (LIA) versus Peripheral Nerve Block of the Femoral and/or Sciatic Nerves (FSNB).

Reference	Study Design	No. of Patients and Group	Main Outcome
Tian et al.^42^	Retrospective	124 LIA 82 FSNB	LIA and FSNB: Similar pain relief outcome
Berninger et al.^43^	Retrospective	76 LIA 58 FSNB	LIA: Better pain relief 24 hours post-op
Berninger et al.^44^	Retrospective	147 LIA 81 FSNB 51 Controls	LIA: Better thigh muscle strength gain
Tanikawa et al.^45^	Prospective double-blinded randomized controlled trial	39 LIA 39 FSNB	FSNB: Better pain relief in first 3 post-op hours
Albrecht et al.^17^	Systematic review and meta-analysis	Not applicable	LIA and FSNB: Similar pain relief and functional outcomes; similar complications

Post-op, postoperative(ly).

## CONCLUSION

This study showed that FSNB provided better pain control than LIA in the immediate post-op hours. However, the short-term enhanced post-op pain relief by FSNB should be weighed against its potential costs, logistics, and the potential for complications that do not exist when using LIA for pain control. Both methods for post-op pain control provide higher pain relief after TKA surgery than standard protocol with pharmacological, parenteral, and oral agents; therefore, they should be of high clinical preference. The decision regarding which methodology to use should be based on the available medical professional’s experience and expertise and be discussed with the patient before surgery.

## Abbreviations

ASA: American Society of Anesthesiologists
FSNB: femoral plus sciatic nerve block
KSS: Knee Society Score
LIA: local infiltration analgesia
post-op: postoperative
ROM: range of motion
TKA: total knee arthroplasty
VAS: visual analog scale.
